# Preterm Birth Causes Decreased Postnatal Neurogenesis: A Novel Mechanism Underlying Adverse Neurodevelopmental Outcomes

**DOI:** 10.1111/ped.70574

**Published:** 2026-10-13

**Authors:** Koya Kawase

**Affiliations:** ^1^ Department of Pediatrics and Neonatology Nagoya City University Graduate School of Medical Sciences Nagoya Aichi Japan; ^2^ Department of Developmental and Regenerative Neurobiology, Institute of Brain Science Nagoya City University Graduate School of Medical Sciences Nagoya Aichi Japan; ^3^ Biotech Research & Innovation Centre (BRIC), Faculty of Health and Medical Sciences, University of Copenhagen Copenhagen Denmark

**Keywords:** neural stem cells, neurodevelopmental disorders, postnatal neurogenesis, preterm birth, ventricular‐subventricular zone

## Abstract

Although the survival rates of preterm infants have markedly improved, their neurodevelopmental outcomes remain poor. Historically, brain injury in preterm infants has been recognized as intraventricular hemorrhage or white matter injury, often resulting in cerebral palsy. More recently, increasing attention has focused on the elevated risk of neurodevelopmental disorders among preterm infants across a wide range of gestational ages. Furthermore, preterm birth is also associated with an increased risk of psychiatric disorders in adulthood, including depression and schizophrenia. However, the underlying pathophysiology of these conditions remains poorly understood, and therapeutic strategies and their efficacy are limited. Preterm birth occurs during a critical period of brain development, when processes including neurogenesis, gliogenesis, and oligodendrocyte maturation are actively progressing. In addition, recent studies have demonstrated that in humans, neural stem cells (NSCs) persist in the ventricular‐subventricular zone (V‐SVZ) during the early postnatal period and continue to generate new neurons. Early postnatal neurogenesis may therefore contribute to brain development during infancy. Although preterm birth may disrupt these developmental processes, its impact on brain development during this critical period remains incompletely understood. We recently discovered that preterm birth causes decreased early postnatal neurogenesis in the V‐SVZ in both mice and humans, and proposed this as a novel mechanism underlying adverse neurodevelopmental outcomes in preterm infants. These findings indicate that targeting postnatal NSC function may provide a new therapeutic approach for neurodevelopmental disorders in preterm infants. Our findings also provide new insights into the mechanisms underlying various long‐term complications of preterm birth.

## 1 Neurodevelopmental Outcomes of Preterm Infants

Preterm birth, defined as a birth before 37 weeks of gestation, remains a major public health issue worldwide. Currently, approximately one in 10 infants is born preterm worldwide, and this rate has not declined between 2010 and 2020 [1]. Recent advances in perinatal medicine have improved the survival rates of preterm infants. In Japan, survival rates are approximately 60% for infants born at 22 weeks of gestation, 80% at 23 weeks, and 90% at 26 weeks [2]. Despite these advances, significant challenges regarding neurodevelopmental outcomes in preterm infants remain unresolved.

Intraventricular hemorrhage (IVH) and white matter injury (WMI) are well‐recognized forms of preterm brain injury [3]. IVH originates from the germinal matrix and extends into the ventricles [4]. The germinal matrix is richly vascularized but is characterized by an immature vascular architecture with fragile vessel walls and limited perivascular support. Consequently, preterm infants are particularly vulnerable to hemorrhage owing to impaired cerebral blood flow autoregulation. Severe IVH can lead to post‐hemorrhagic hydrocephalus or intraparenchymal hemorrhage secondary to venous infarction, resulting in cerebral palsy and neurodevelopmental delay [5].

WMI encompasses non‐hemorrhagic white matter injuries in preterm infants [6]. Historically, WMI has been described as periventricular leukomalacia (PVL) [7]. However, advances in magnetic resonance imaging (MRI) have revealed that WMI in preterm infants is more heterogeneous than previously recognized. Consequently, Dr. Joseph J. Volpe, a leading authority in neonatal neurology, has suggested that the term “PVL” is no longer appropriate [8]. The underlying pathogenesis of WMI involves intrinsic vulnerabilities of the preterm brain, which are exacerbated by extrinsic insults such as hypoxia, ischemia, and inflammation. These insults lead to the death and maturation arrest of preoligodendrocytes, resulting in impaired myelination [9]. In addition, WMI may be accompanied by axonal injury and neuronal loss in the thalamus, basal ganglia, and cerebral cortex [10]. These complex pathologies go beyond mere WMI and have been described by Volpe as “encephalopathy of prematurity” [11]. Severe WMI is associated with cerebral palsy and neurodevelopmental delays [12].

Even in the absence of IVH or WMI, the neurodevelopmental outcomes of preterm infants are not necessarily favorable. Recent studies have shown that preterm infants without severe IVH or WMI may have adverse neurodevelopmental outcomes [13]. Attention has also been focused on late preterm infants born at 34–36 weeks of gestation, who typically do not present with IVH or WMI but nevertheless demonstrate poorer neurodevelopmental outcomes than term infants [14, 15, 16]. Recently, increasing attention has focused on the elevated risk of neurodevelopmental disorders (NDDs) among preterm infants across a wide range of gestational ages [17, 18]. These findings suggest that preterm birth, even in the absence of overt brain injury, can have long‐term effects on brain development, particularly affecting multiple neurocognitive and behavioral domains. However, the underlying pathophysiological mechanisms remain unclear.

Moreover, recent evidence has indicated that preterm birth is associated with an increased risk of psychiatric disorders in adulthood, including depression and schizophrenia [19, 20, 21]. Adult psychiatric disorders, like childhood NDDs, substantially impair the quality of life of affected individuals and their families and impose considerable social and economic burdens [22]. However, the mechanisms by which preterm birth contributes to the development of psychiatric disorders in adulthood also remain unclear.

## 2 Neurodevelopmental Disorders in Preterm Infants

Advances in perinatal care have led to a reduction in the incidence of IVH and severe WMI worldwide [23]. Therefore, the contemporary neurological sequelae of preterm birth are shifting from cerebral palsy secondary to IVH/WMI toward NDDs observed across a wide range of gestational ages.

NDDs are a group of conditions arising from abnormalities in the development of the central nervous system from the fetal period through early childhood. They exhibit diverse clinical manifestations that affect motor function, cognitive abilities, language and communication skills, attention and behavior, and social development [24, 25, 26]. Representative NDDs include attention‐deficit/hyperactivity disorder (ADHD), autism spectrum disorder (ASD), learning disorders (LD), intellectual disability, developmental coordination disorder, and language disorders. While each of these disorders has specific clinical features, symptoms, and functional impairments often overlap.

Multiple large‐scale cohort studies have shown that the risk of developing ADHD in preterm infants increases proportionally with decreasing gestational age [27, 28, 29]. In a large Swedish cohort, the prevalence of ADHD was 4.5% in full‐term infants (39–41 weeks), 12.1% in extremely preterm infants (22–27 weeks), 7.0% in moderately preterm infants (28–33 weeks), and 5.6% in late preterm infants (34–36 weeks) [27]. Extremely preterm infants had more than twice the risk of ADHD compared with full‐term infants. Interestingly, even early‐term infants (37–38 weeks) exhibited a significantly increased risk compared with full‐term infants. These findings suggest that even a modest shortening of gestational age can have measurable effects on neurodevelopment during childhood. These patterns persisted after adjusting for sociodemographic and perinatal factors as well as familial confounding, indicating a robust association between shortened gestation and ADHD risk, independent of genetic or environmental influences [27].

Moreover, multiple large‐scale epidemiological studies have also consistently reported that preterm infants have a significantly higher risk of developing ASD [24, 30]. In a nationwide Swedish birth cohort study, the prevalence of ASD was 1.4% among full‐term infants (39–41 weeks), 6.1% among extremely preterm infants (22–27 weeks), 2.1% among all preterm infants (< 37 weeks), and 1.6% among early‐term infants (37–38 weeks). Extremely preterm infants had an approximately four‐fold higher risk of ASD than full‐term infants [24]. Importantly, this association persisted even after adjusting for shared familial factors, suggesting that gestational age itself is a strong independent risk factor [24].

Preterm infants have also been shown to have a higher risk of developing LD compared with term infants [31, 32]. LD is an NDD characterized by difficulties in specific learning domains, such as reading, arithmetic, and writing, which can have long‐term effects on academic performance and learning abilities during school age. In a U.S. cohort study, all preterm infants (< 37 weeks) were found to have approximately 1.5‐fold increased risk of LD compared with term infants [32]. In addition, a meta‐analysis integrating multiple cohort studies showed that preterm children continue to exhibit lower academic performance in reading and mathematics during school age [33].

In addition to ADHD, ASD, and LD, preterm infants are at an increased risk of a range of other neurodevelopmental difficulties such as deficits in attention, executive function, motor skills, and social–emotional development [34, 35, 36]. Interventions for NDDs in preterm infants primarily focus on early developmental support and educational assistance, as no specific treatment currently exists.

## 3 Functional Roles and Developmental Vulnerability of the Prefrontal Cortex

The prefrontal cortex (PFC) is the most highly evolved region of the human cerebral cortex and serves as a central hub for complex higher brain functions, including executive function, working memory, attentional control, decision‐making, emotional regulation, and social cognition [37]. By integrating sensory input and emotional information, the PFC enables context‐appropriate behavioral selection and inhibition, thereby supporting goal‐directed behavior and social adaptation. Owing to these functional characteristics, the PFC plays a pivotal role in understanding both neurodevelopmental processes and the pathophysiology of psychiatric disorders [38].

NDDs are strongly associated with dysfunction of PFC. In ADHD, core clinical features such as impaired attentional control, impulsivity, and hyperactivity are linked to immaturity and functional deficits within executive control networks centered on the dorsolateral PFC and anterior cingulate cortex [39, 40]. Neuroimaging studies have demonstrated reductions in PFC thickness, delayed cortical maturation, and altered connectivity within frontostriatal circuits [41]. In ASD, deficits in social interaction, reduced behavioral flexibility, and repetitive behaviors have been associated with dysfunction of networks connecting the PFC with the temporal and limbic regions [42]. In particular, the medial PFC and orbitofrontal cortex, which are critical for social cognition and value‐based decision‐making, consistently exhibit structural and functional abnormalities in individuals with ASD [43].

Abnormalities of the PFC are not confined to childhood NDDs, but are also closely implicated in psychiatric disorders that emerge in adulthood. In major depressive disorder, reduced activity and volume of the dorsolateral and ventromedial PFC have been reported and are believed to underlie impairments in emotional and cognitive regulation [44, 45]. The PFC is also a central site of schizophrenia pathology [46, 47]. Recent single‐nucleus RNA‐sequencing studies have revealed transcriptional abnormalities in cortical projection neurons and GABAergic interneurons within the PFC, particularly involving gene networks related to synapse formation, mitochondrial function, and translational regulation [48, 49]. These molecular findings support the neurodevelopmental hypothesis that vulnerabilities established within PFC circuits during development may interact with environmental factors after adolescence to manifest as psychiatric illnesses.

MRI studies have reported reductions in the volume and surface area of the PFC in preterm infants throughout childhood and adolescence [50, 51, 52, 53]. Thus, brain dysfunction in preterm infants may reflect impaired PFC development and the subsequent dysmaturation of cortical neuronal networks, particularly in the contemporary era.

## 4 Preterm Birth Disrupts Cortical Interneuron Development

Cortical interneuron development continues during late gestation and may therefore be particularly vulnerable to preterm birth. In a preterm rabbit model, preterm birth was associated with a transient increase in Nkx2.1^+^, Dlx2^+^, and Sox2^+^ interneuron progenitors in the medial ganglionic eminence, suggesting an alteration in the timing of interneuron neurogenesis. This increase was associated with premature withdrawal of maternal estrogen, as estrogen treatment reduced the expansion of interneuron progenitors [54]. Alterations in interneuron subtype composition have also been observed in the cerebral cortex. Human preterm brains showed reduced densities of PV^+^ and GAD67^+^ interneurons together with an increased density of SST^+^ interneurons in the upper cortical layers, suggesting that preterm birth may alter the composition and maturation of cortical interneuron subtypes. These changes were partially reversed by estrogen treatment in a preterm rabbit model, with increased PV^+^ and transiently reduced SST^+^ interneurons [55]. In addition to changes in interneuron number and subtype composition, their morphological maturation may also be affected. Human preterm brains with diffuse WMI showed reduced numbers of calretinin^+^ interneurons and impaired neurite growth and branching of SST^+^ and NPY^+^ interneurons. In a mouse model, early‐life inflammation similarly disrupted the development of PV^+^ interneurons and their association with perineuronal nets [56].

Together, these findings suggest that preterm birth can affect cortical interneurons at multiple stages, from progenitor production to subtype composition and neuronal maturation. Such alterations may disturb the balance between excitatory and inhibitory signaling and contribute to abnormal cortical circuit development following preterm birth.

## 5 Postnatal Neurogenesis in the Ventricular‐Subventricular Zone

For many years, it was believed that neurons are not generated in the postnatal brain [57]. However, since the 1990s, accumulating evidence has demonstrated that neural stem cells (NSCs) are retained in a specific region of the postnatal brain, the ventricular‐subventricular zone (V‐SVZ), and that neurogenesis continues after birth in mammals, including humans [58] (Figure 1). The postnatal NSC niche, V‐SVZ, is composed of several cell types. The ventricular surface is covered by ependymal cells, which have motile cilia on their apical surfaces. Postnatal NSCs are surrounded by multiple ependymal cells and positioned at the center, forming a characteristic structure known as the pinwheel architecture [59]. Postnatal NSCs are activated by signaling molecules, such as BMP5, IGF, and FGF2, leading to the generation of intermediate progenitor cells [60, 61, 62]. These intermediate progenitor cells undergo three to four rounds of division before differentiating into neurons [63]. In rodents, neurons generated in the V‐SVZ migrate to the olfactory bulb (OB), the primary center of olfaction, via a specialized migratory pathway known as the rostral migratory stream (RMS) [58, 64, 65]. They form chain‐like cellular aggregates within the RMS [58, 66]. Upon reaching the OB, newborn neurons predominantly differentiate into inhibitory interneurons that are involved in the modulation of various olfactory functions [58, 67].

**FIGURE 1:**
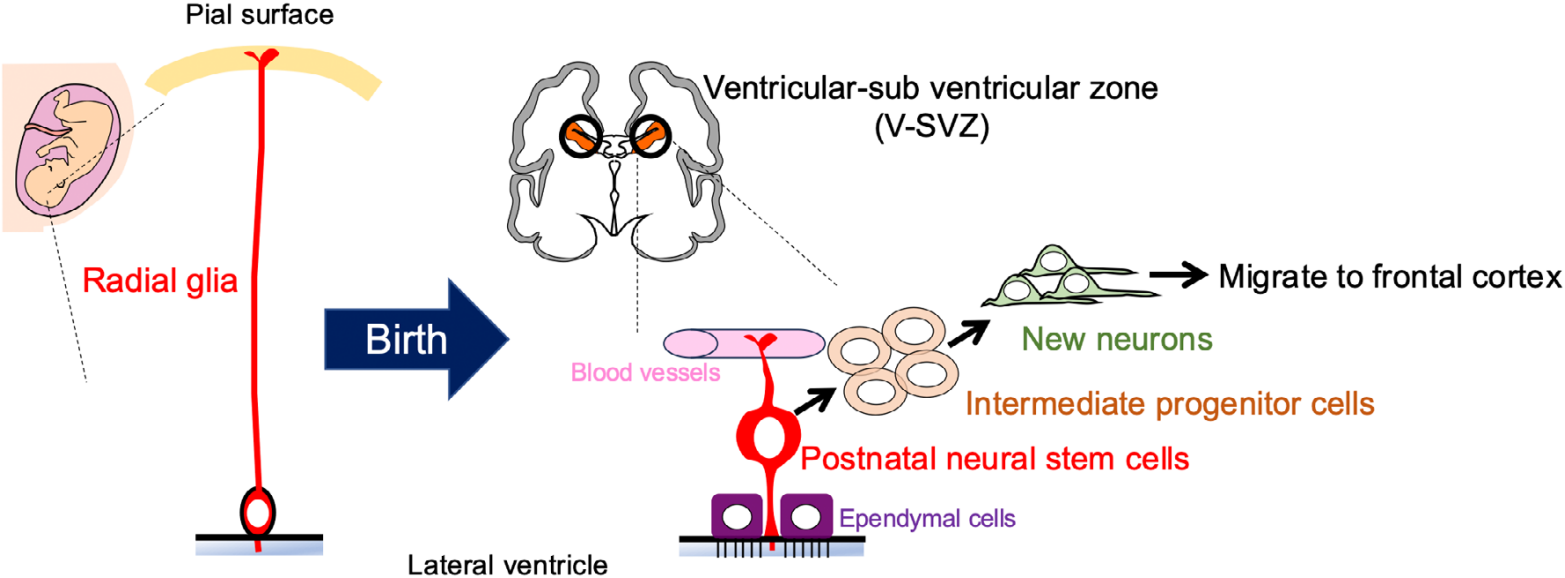
Schema of the neural stem cell niche. In the fetal brain, radial glia act as neural stem cells and extend long thin fibers to the pial surface. After birth, radial glia undergo morphological remodeling, characterized by the shortening of their fibers, and transform into postnatal NSCs in the ventricular‐subventricular zone. The distal ends of these postnatal NSCs form endfeet that contact blood vessels.

Interestingly, in human neonatal and infantile brains, it has been reported that new neurons generated in the V‐SVZ migrate not only to the OB, but also to the ventral medial PFC through a migratory pathway known as the medial migratory stream [68, 69]. More recently, these neurons were shown to migrate broadly into the frontal cortex during the neonatal period and early infancy [70]. This migratory stream, termed the “Arc,” is characterized by clusters enriched in Dcx^+^ new neurons [70]. Immunohistochemical analyses revealed that Dcx^+^ cells around the walls of the lateral ventricles form a four‐tiered structure: Dcx^+^ cells along the ventricular wall (Tier 1), dispersed cells away from the ventricular wall (Tier 2), cells located around blood vessels (Tier 3), and clusters of cells oriented toward the pia in the developing white matter (Tier 4) [70]. Dcx^+^ cells in the Arc express GABA, GAD67, and CXCR4, exhibiting characteristics of the inhibitory interneuron lineage [70]. V‐SVZ neurogenesis persists into adulthood in rodents, whereas it is known to decline in humans and largely disappears by approximately 18 months of age [68]. The migration of V‐SVZ‐derived new neurons toward the frontal cortex is reported to be most prominent at approximately 1.5 months of age and to be almost undetectable by 2 years of age. After migrating to the frontal cortex, these cells are thought to mature into interneurons expressing SST, NPY, CalB, Nkx2.1, COUP‐TFII, and Sp8 [70]. These observations suggest that early postnatal neurogenesis in humans plays a critical role in the development of the frontal cortex during the neonatal and infant period, and may be important for the acquisition of multiple neurocognitive and behavioral functions. We previously found that when brain injury occurs, new neurons generated in the V‐SVZ migrate toward the injured area, where a subset replaces the lost neurons [71, 72]. Therefore, postnatal neurogenesis in the V‐SVZ may contribute to neuronal regeneration after brain injury.

Postnatal NSCs originate from radial glia (RG) during embryonic development [73] (Figure 1). RG extend thin fibers from the soma, located in the ventricular zone, to the pial surface [74, 75]. During brain development, these fibers act as a “scaffold” for newly generated neurons derived from the RG to migrate toward the pial surface, playing a crucial role in positioning neurons at their proper locations [74, 76]. RG are not restricted to neuronal production but can also generate glial cells during later stages of development. Early studies demonstrated that a population of RG in the developing primate brain undergoes a transition toward astrocytic differentiation, providing evidence that RG contribute substantially to astrocyte production [77]. After birth, the RG undergo shortening of these fibers, and their distal ends form endfeet that contact the blood vessels [59, 78] (Figure 1). As a result, the RG transform into postnatal NSCs. The RG also differentiate into ependymal cells after birth [79]. Thus, NSCs undergo dynamic changes in their morphology and fate associated with birth. In addition, recent studies have expanded our understanding of the gliogenic potential of human RG. In particular, outer RG, which are abundant in the developing human outer subventricular zone, have been shown to generate oligodendrocyte precursor cells (OPCs), providing an additional source of cortical OPCs during human development [80]. In humans, RG persist until the third trimester, and it has been reported that these cells, as in mice, transform into postnatal NSCs after birth [81, 82].

During the embryonic period, RG are in an activated state characterized by active proliferation and robust production of intermediate progenitor cells. After birth, RG acquire quiescence, thereby preventing the depletion of the postnatal NSC pool and enabling long‐term postnatal neurogenesis [83, 84]. However, the mechanisms through which NSCs acquire quiescence after birth have not been fully elucidated. The effects of preterm birth on NSC function and early postnatal neurogenesis are poorly understood.

## 6 Effects of Birth‐Induced Metabolic Changes on Radial Glia

Birth is one of the most important life events. The transition from an intrauterine to an extrauterine environment causes dynamic metabolic changes. Oxygen metabolism is the most critical metabolic change induced during birth. Fetal arterial oxygen tension (PaO_2_) in utero is approximately 20–30 mmHg, which is markedly lower than the PaO_2_ of 60–80 mmHg observed in neonates [85]. In other words, the fetus develops in a hypoxic environment. This raises the question of how the partial pressure of oxygen (pO_2_) in the NSC niche changes before and after birth. Importantly, the relationship between oxygen tension and NSC fate has been extensively investigated. Under hypoxic conditions, NSC self‐renewal is promoted through HIF‐1α signaling, whereas increasing oxygen levels have been shown to promote differentiation toward neuronal and glial lineages [86, 87, 88].

To investigate the effects of birth on NSCs in the V‐SVZ, we used normal term mice and mifepristone‐induced preterm birth mice. In this preterm birth mouse model, pups were delivered just 1 day before the expected date of birth without the induction of any exogenous hypoxia, ischemia, or inflammation [89]. We found that the pO_2_ levels in the mouse V‐SVZ increased with birth. Notably, in preterm birth mice, pO_2_ levels in the V‐SVZ also increased to levels comparable to those in term birth mice [90]. Furthermore, single‐cell RNA sequencing of the mouse V‐SVZ revealed that during normal term birth, the expression of glycolysis‐related genes, including *Eno1*, *Gapdh*, and *Ldha*, was downregulated in RG. These genes are upregulated under hypoxic conditions [91]. Thus, an increase in pO_2_ levels in the V‐SVZ at birth appears to contribute to the downregulation of glycolysis‐related genes in RG. Interestingly, in preterm birth mice, the downregulation of glycolysis‐related genes in RG was incomplete. Preterm birth may impair mitochondrial function, rendering RG unable to fully utilize oxygen despite the rise in pO_2_ levels in the V‐SVZ.

Glutamine metabolism also plays a critical role in regulating NSC activation. Glutamine is the most abundant and versatile amino acid in the body, and its metabolism is essential for tissue homeostasis. Glutamine is catabolized to glutamate by glutaminases (Gls) and can enter the tricarboxylic acid cycle after conversion to α‐ketoglutarate through a process called glutaminolysis (Figure 2). Glutaminolysis is a hallmark of mitotically active cells, especially tumor cells [92]. Recent studies have shown that glutaminolysis is essential for embryonic NSCs [93, 94]. Conversely, glutamine is synthesized from glutamate by glutamate‐ammonia ligase (Glul), also known as glutamine synthetase (GS) (Figure 2). We found that *Gls* was barely expressed in RG. *Glul* is largely not expressed in ependymal cells, intermediate progenitor cells, OPCs, or neurons in the V‐SVZ, but is preferentially expressed in RG. These results suggest that glutamine metabolism in RG was primarily regulated by Glul. Following term birth, Glul expression in RG was upregulated, indicating that birth induces active synthesis of glutamine from glutamate in RG. Interestingly, in preterm birth mice, this birth‐induced upregulation of Glul in RG was incomplete, suggesting that birth‐induced alteration of glutamine metabolism in RG is impaired by preterm birth [90].

**FIGURE 2:**
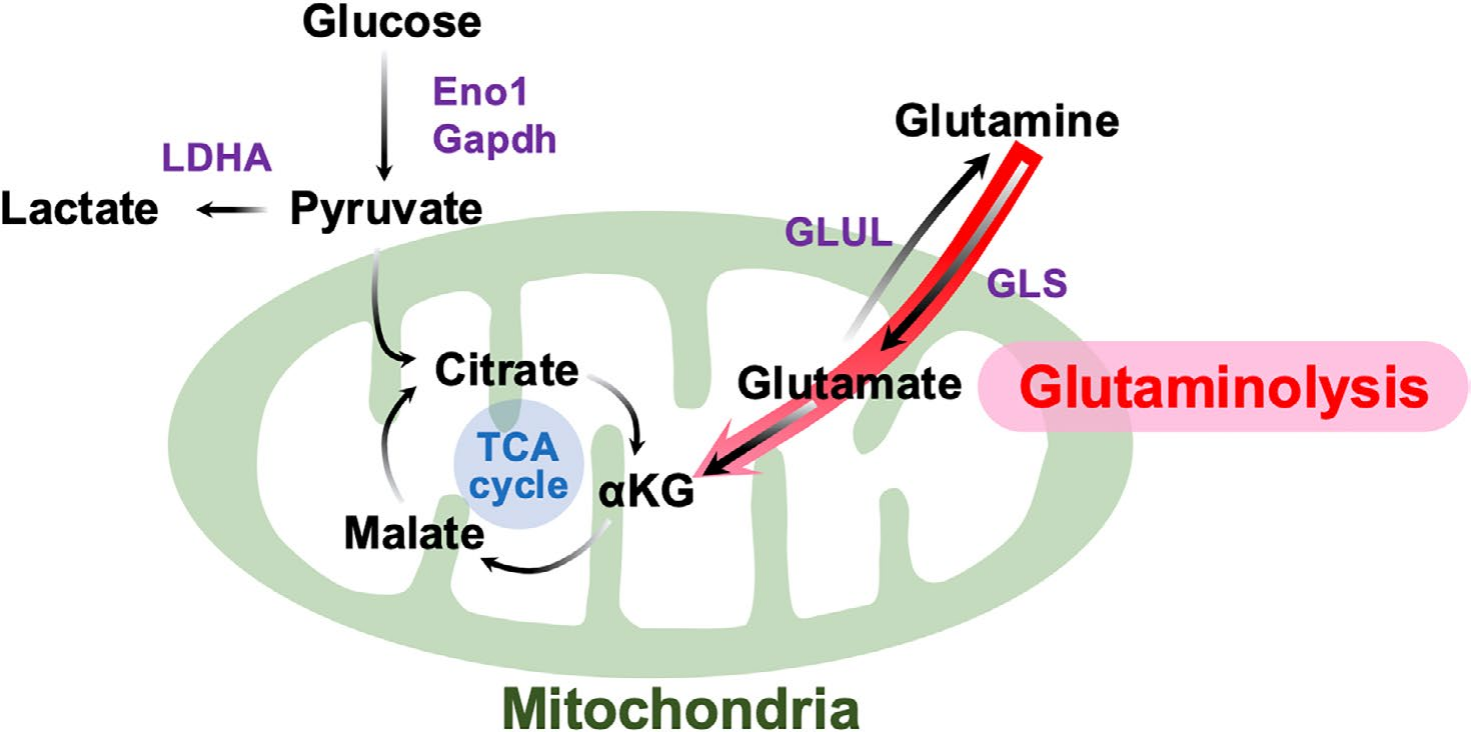
Schema of glutamine metabolism. Glutamine is catabolized to glutamate by glutaminases (Gls) and can enter the tricarboxylic acid cycle after conversion to α‐ketoglutarate through a process called glutaminolysis. Glutamine is synthesized from glutamate by the glutamate‐ammonia ligase (Glul).

Gene set enrichment analysis further revealed that the quiescent stem cell signature was enriched in RG of term mice at postnatal Day 2 (P2) compared with the level in RG at embryonic Day 18.5 (E18.5). When comparing preterm mice at P0 with E18.5 fetuses, which correspond to the same days post‐coitus, the quiescent stem cell signature was also enriched in preterm P0 RG compared with the level in E18.5 RG. This suggests that, as a consequence of the earlier birth, the transition toward RG quiescence begins earlier in preterm mice than in term mice. However, when preterm mice at P3 were compared with term mice at P2, which also correspond to the same days post‐coitus, there was a tendency for the quiescent stem cell signature to be suppressed and the active stem cell signature to be enriched in preterm P3 RG compared with the level in term P2 RG. These results suggest that birth triggers a transition of RG from an active to a quiescent state, but that the transition in gene expression related to these stem cell signatures is impaired by preterm birth [90]. Moreover, preterm birth accelerates the shortening of the RG process, indicating that birth directly drives the morphological conversion of RG into postnatal NSCs [95].

## 7 Preterm Birth Causes Decreased Early Postnatal Neurogenesis

mTORC1 signaling is known to promote NSC activation [96]. In term mice, mTORC1 signaling in RG is downregulated after birth, whereas it is transiently upregulated in preterm mice [90]. Preterm mice exhibit a temporary increase in Mash1^+^ intermediate progenitor cell production in the lateral V‐SVZ. However, at the young‐adult stage, they exhibit a decrease in activated NSCs, accompanied by a reduction in Mash1^+^ intermediate progenitor cells and Dcx^+^ new neurons in the lateral V‐SVZ [90]. To investigate whether the effects of preterm birth differed among V‐SVZ regions, we divided the lateral V‐SVZ into three regions: dorso‐lateral, mid‐lateral, and ventral [90]. The number of Mash1^+^ intermediate progenitor cells was decreased in all three regions, suggesting that the reduction in Mash1^+^ intermediate progenitor cells occurred uniformly throughout the lateral V‐SVZ following preterm birth [90]. The number of Dcx^+^ new neurons did not differ between term and preterm mice in the dorsal or medial V‐SVZ. The Mash1^+^ intermediate progenitor cells in the V‐SVZ are neuronal progenitor cells that differentiate into Dcx^+^ newborn neurons, which then migrate to the granule cell layer (GCL) of the OB and mature into granule cell interneurons [97]. Importantly, we found the reduction of NeuN^+^ mature neurons in the GCL of the OB in preterm mice [90]. We also observed that NSCs in preterm birth mice displayed impaired endfoot formation with vasculature and abnormal morphology [95]. Previous studies have shown that endothelial cells provide niche signals that promote NSC self‐renewal and help maintain NSCs in a primitive state [98, 99]. Thus, the impaired endfoot formation with the vasculature observed after preterm birth may disrupt NSC‐endothelial interactions and associated niche signals, potentially contributing to impaired NSC maintenance and stemness.

Furthermore, we used *GFAP‐EGFP* mice to evaluate the number of NSCs at the young‐adult stage. As NSCs express GFAP, they are labeled with EGFP. EGFP^+^ cells with apical contacts at the lateral ventricle were defined as NSCs. Among these NSCs, cells expressing both EGFR and Mash1, which are markers of active and neurogenic states, were defined as active and neurogenic NSCs. We found that the number of active and neurogenic NSCs was reduced in preterm mice compared with term mice at the young‐adult stage [90]. We further used the *GFAP‐CreERT2;R26‐tdTomato* double‐transgenic mouse line, in which tamoxifen treatment induces tdTomato expression in GFAP^+^ NSCs and their progeny. This approach allowed us to trace the fate of NSCs and the cells generated from them, enabling us to assess the capacity of NSCs to generate intermediate progenitor cells. At 5 days after tamoxifen administration, the ratio of tdTomato^+^Mash1^+^ intermediate progenitor cells to tdTomato^+^GFAP^+^ NSCs was decreased in preterm mice, suggesting a reduced capacity of NSCs to generate intermediate progenitor cells [90]. In contrast, we found no difference in the proliferative capacity of intermediate progenitor cells between term and preterm mice. Taken together, these findings suggest that the transient hyperactivation of RG during the early postnatal period following preterm birth leads to a reduction in active and neurogenic NSCs at the young‐adult stage, resulting in impaired neurogenesis. As described above, the decreased postnatal neurogenesis following preterm birth is observed in the lateral V‐SVZ, suggesting that preterm birth may deplete NSCs in this region.

We also analyzed the effect of preterm birth on V‐SVZ neurogenesis in human preterm infants. For this purpose, fixed postmortem brains from full‐term infants born at ≥ 37 weeks of gestation (*n* = 3) and preterm infants born at ≤ 34 weeks of gestation (*n* = 3) were analyzed. All cases died at 0–3 months corrected age. Analysis of human postmortem brains revealed a similar pattern to that observed in mice, with a reduction of Dcx^+^ new neurons in the V‐SVZ of preterm infants [90] (Figure 3). In addition, the number of clusters of Dcx^+^ neurons in the V‐SVZ is decreased in preterm infants [95]. These findings indicate that preterm birth leads to a decrease in early postnatal neurogenesis in mice and humans. Considering that V‐SVZ‐derived neurons in humans migrate to the PFC [68, 70], decreased early postnatal neurogenesis may contribute to worse neurodevelopmental outcomes in preterm infants, particularly cognitive dysfunction and impaired social behavior.

**FIGURE 3:**
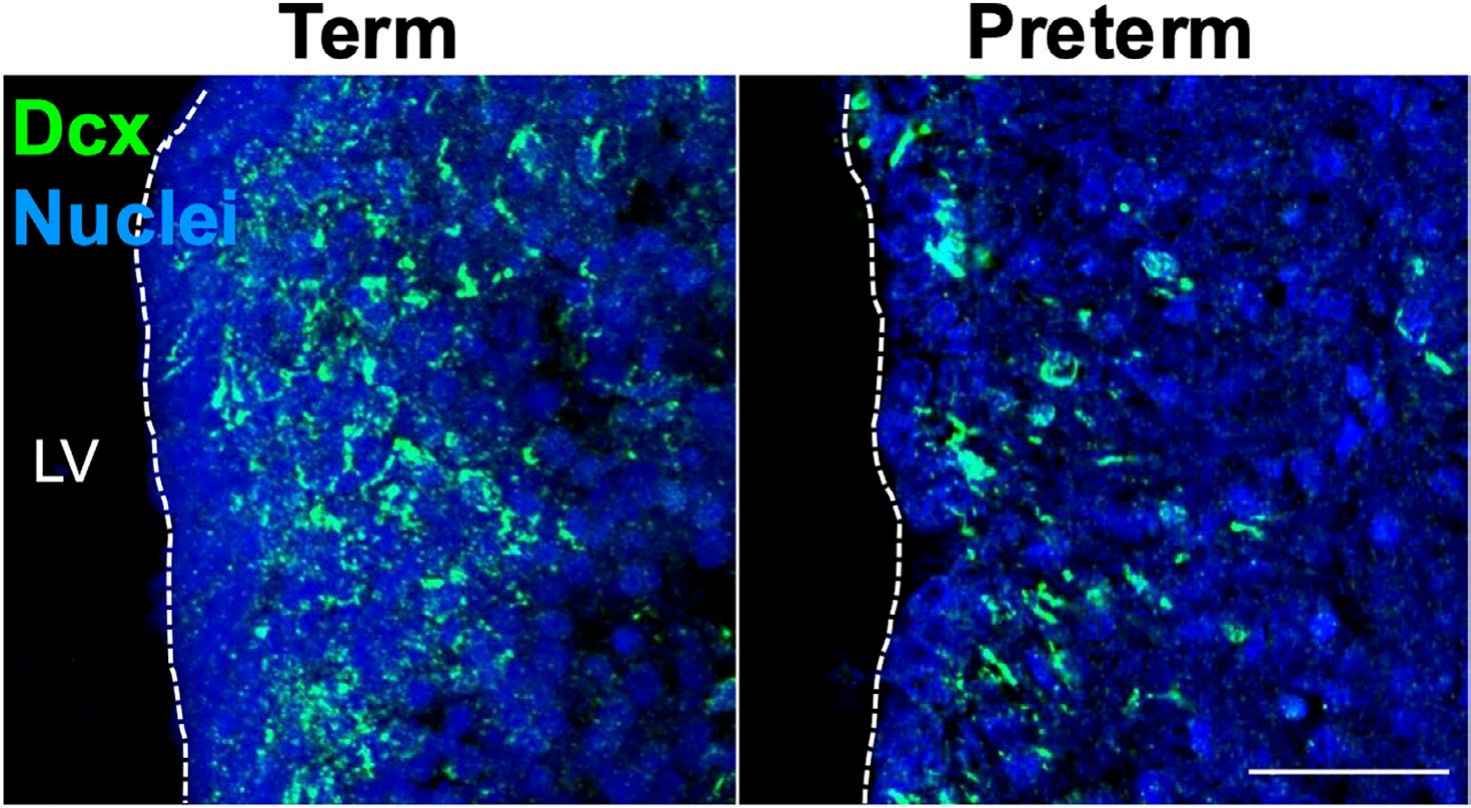
Decreased early postnatal neurogenesis in the V‐SVZ of preterm infants. Representative images of coronal V‐SVZ sections of the human brain stained with Dcx (green). Nuclei were stained with DAPI. Scale bar, 50 μm. LV, lateral ventricle.

Other studies have shown that perinatal hypoxia, ischemia, and inflammation affect V‐SVZ cells. Analyses of autopsied brains from preterm infants born at 23–27 weeks of gestation with pathologically confirmed WMI revealed irregularities in the ventricular surface and disruption of cellular organization in the V‐SVZ [100]. Furthermore, a recent study using a mouse model of intestinal perforation reported that inflammation spreading to the V‐SVZ induces the death of ependymal cells, thereby impairing the chain migration of new neurons [101]. It is known that the coordinated beating of multiciliated ependymal cells drives cerebrospinal fluid (CSF) flow, which generates concentration gradients that guide directional neuronal migration in the adult mammalian brain [102]. Therefore, the loss of ependymal cells may disrupt CSF flow and consequently impair the chain migration of newborn neurons derived from the V‐SVZ. However, the possibility that systemic inflammation may impair chain migration independently of ependymal cell loss cannot be excluded. In contrast, our study demonstrates that preterm birth itself leads to decreased early postnatal neurogenesis in the V‐SVZ.

## 8 Birth‐Induced Alteration of Glutamine Metabolism Is Required for the Acquisition of Quiescence of Neural Stem Cells

In the embryonic brain, RG actively proliferate and generate neurons despite residing in a hypoxic intrauterine environment. What mechanisms allow this? Embryonic RG exhibit low Glul expression, which may activate glutaminolysis. In addition, the abundant supply of amino acids from the placenta provides a favorable environment for the utilization of glutamine as a substrate for active glutaminolysis. Following birth, the RG exit the hypoxic intrauterine environment, and the supply of amino acids from the placenta is disrupted. Considering that glutaminolysis is typically active in hypoxic cells, birth‐induced upregulation of Glul in RG may represent a response to exit from the low‐oxygen uterine environment. Glul upregulation appears to suppress glutaminolysis. We further demonstrated that the upregulation of Glul in RG induces quiescence acquisition via the downregulation of mTORC1 signaling [90] (Figure 4). Interestingly, high expression of *Glul* in adult quiescent NSCs has also been reported [103], suggesting that Glul‐dependent regulation of NSC activity occurs in both neonates and adults.

**FIGURE 4:**
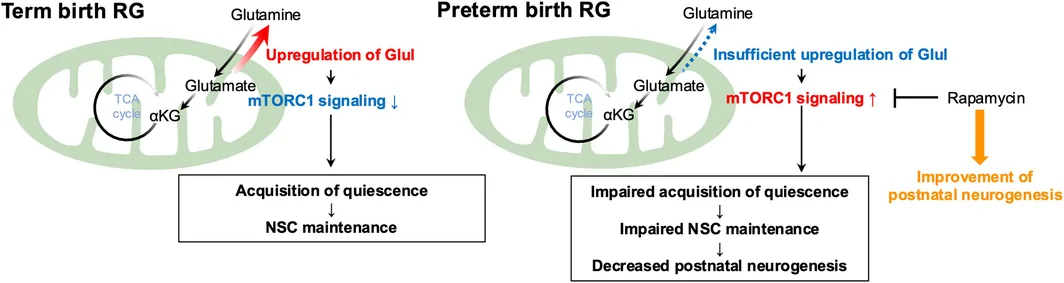
Summary of our study. Left: At term birth, Glul expression in RG is upregulated, leading to the downregulation of mTORC1 signaling. As a result, RG acquire quiescence, ensuring long‐term maintenance of the postnatal NSC pool. Right: In preterm births, the postnatal upregulation of Glul in RG is incomplete. Consequently, mTORC1 signaling is transiently hyperactivated, leading to the impaired acquisition of quiescence in RG. Consequently, preterm birth depletes the NSC pool and decreases postnatal neurogenesis. The administration of rapamycin, an mTORC1 inhibitor, restored quiescence in RG after preterm birth, thereby preventing NSC depletion and improving postnatal neurogenesis.

In preterm birth, RG fail to adapt appropriately to exit from the hypoxic intrauterine environment, resulting in insufficient upregulation of Glul. Consequently, mTORC1 signaling in RG is transiently upregulated, impairing quiescence. This leads to depletion of the NSC pool and decreased postnatal neurogenesis at the young‐adult stage. Importantly, the inhibition of excessive mTORC1 signaling with rapamycin in preterm birth mice improved postnatal neurogenesis (Figure 4), suggesting a potential therapeutic strategy targeting NSC function in the V‐SVZ to treat NDDs in preterm infants [90].

We also demonstrated that the inhibition of the postnatal increase in oxygen prevented RG fiber shortening, suggesting that the transition from a hypoxic intrauterine environment to an oxygen‐rich extrauterine environment is a critical factor in NSC morphological remodeling [95]. Given that direct contact between NSC and vascular endothelial cells is essential for maintaining NSC quiescence [98, 104], impaired endfoot formation in RG due to preterm birth may also contribute to the impaired acquisition of quiescence.

Whether the decrease in early postnatal neurogenesis caused by preterm birth is mediated by the same mechanisms in humans and mice remains unclear. In preterm infants, numerous factors, beyond alterations in oxygen availability and glutamine metabolism, may influence brain development. These include respiratory and circulatory instability and related medical interventions, hypoglycemia, repeated exposure to painful medical procedures, prolonged maternal–infant separation, and diverse hormonal fluctuations. Whether and how these factors affect early postnatal neurogenesis requires further investigation. Additionally, it remains unclear whether the decreased early postnatal neurogenesis in the V‐SVZ caused by preterm birth leads to a decrease in the number of neurons in the PFC. Furthermore, the effects of preterm birth on the functional properties of prefrontal cortical neurons and the mechanisms by which such alterations contribute to the development of NDDs and psychiatric illnesses in adulthood remain largely unknown.

## 9 Quiescence of Neural Stem Cells Induced by Environmental Shifts: Evolutionary Perspectives

Postnatal neurogenesis in the V‐SVZ is widely conserved across vertebrates [105, 106]. However, the potential for neurogenesis throughout life varies considerably among animal classes, and the underlying regulatory mechanisms remain incompletely understood. In zebrafish, which spend their entire life in water, a relatively hypoxic environment, RG continue to cover the surface of the adult telencephalic ventricle and maintain a high neurogenic potential throughout life [107, 108]. This condition is reminiscent of the mammalian fetus, which develops in a physiologically hypoxic intrauterine environment. In contrast, amphibians undergo a dramatic transition from water to land during metamorphosis, which results in exposure to elevated oxygen levels. Correspondingly, the proliferative potential of RG declines as tadpoles mature into frogs [109]. This mirrors the postnatal changes observed in mammals, where birth exposes NSCs to an oxygen‐rich environment, triggering metabolic and morphological transitions that promote quiescence. Considering that glutaminolysis is activated under hypoxic conditions [110, 111], it is plausible that RG stemness is maintained through hypoxia‐dependent glutaminolysis in vertebrates.

From an evolutionary perspective, the acquisition of NSC quiescence in terrestrial vertebrates, including birds and mammals, may represent an adaptive strategy to balance regenerative capacity with the long‐term maintenance of the NSC pool. In contrast, aquatic vertebrates retain robust neurogenic and regenerative potential throughout their lives, likely reflecting their persistently hypoxic habitats. Thus, the interplay between environmental oxygen, metabolic regulation, and NSC quiescence may be a critical factor shaping the diversity of regenerative capacities observed across vertebrate evolution.

## 10 Conclusion

We demonstrated that the proper timing of birth is essential for NSCs to acquire quiescence and long‐term maintenance [90]. Preterm birth impairs this cellular process, leading to depletion of the NSC pool. Various tissues in the body harbor tissue‐specific stem cells, and preterm birth may compromise their maintenance. These impairments can disrupt tissue homeostasis and contribute to various long‐term complications in preterm infants.

We propose that decreased early postnatal neurogenesis represents a novel pathogenic mechanism underlying adverse neurodevelopmental outcomes in preterm infants and may contribute to NDDs. With improvements in the survival of preterm infants, the number of individuals transitioning from childhood to adolescence and adulthood has increased worldwide. Our study provides insights into the etiology of brain dysfunction in preterm infants and, more broadly, into the processes underlying the acquisition of higher brain functions in humans. Targeting early postnatal neurogenesis may offer a promising strategy for developing interventions to improve neurodevelopmental outcomes in preterm infants.

## Author Contributions

K.K. wrote and approved the final manuscript.

## Funding

This work was supported by JSPS KAKENHI (23K14983, 25KK0082) (to K.K.) and The Nitto Foundation (to K.K.).

## Ethics Statement

This study was conducted in accordance with the Declaration of Helsinki and was approved by the appropriate institutional ethics committee.

## Conflicts of Interest

The author declares no conflicts of interest.

## Data Availability

The data that support the findings of this study are available from the corresponding author upon reasonable request.
